# Norovirus GII.4/Sydney/2012 in Italy, Winter 2012–2013

**DOI:** 10.3201/eid1908.130619

**Published:** 2013-08

**Authors:** Giovanni M. Giammanco, Simona De Grazia, Fabio Tummolo, Floriana Bonura, Adriana Calderaro, Alessio Buonavoglia, Vito Martella, Maria C. Medici

**Affiliations:** Università degli Studi di Palermo, Palermo, Italy (G.M. Giammanco, S. De Grazia, F. Bonura);; Università degli Studi di Parma, Parma, Italy (F. Tummolo, A. Calderaro, M.C. Medici);; Medical Practitioner, Bari, Italy (A. Buonavoglia);; Università Aldo Moro di Bari, Valenzano, Italy (V. Martella)

**Keywords:** norovirus, surveillance, Italy, GII.4, variant Sydney 2012, viruses, enteric infections, gastroenteritis, genogroups

**To the Editor:** Noroviruses (NoVs) are the major cause of acute gastroenteritis in children and adults; they are responsible for sporadic cases and outbreaks of gastroenteritis in various epidemiologic settings. NoVs can be classified genetically into at least 5 genogroups, GI to GV (*1*). Although >30 genotypes within genogroups GI, GII, and GIV can infect humans (*2*), a single genotype, GII.4, has been associated with most NoV-related outbreaks and sporadic cases of gastroenteritis worldwide (*3*).

GII.4 NoV strains continuously undergo genetic/antigenic diversification and periodically generate novel strains through accumulation of punctate mutations or recombination. New GII.4 variants emerge every 2–3 years (*4*). Increased incidence of NoV-related illness and/or outbreaks in various countries in late 2012 has been related to the emergence of a novel GII.4 variant, Sydney 2012. This variant was first identified in March 2012 in Australia (*5*).

The Italian Study Group for Enteric Viruses (ISGEV; http://isgev.net) monitors the epidemiology of enteric viruses in children through hospital-based surveillance (*6*–*8*). NoVs are monitored and characterized by multitarget analysis in the diagnostic regions A (open reading frame 1, polymerase) and C (open reading frame 2, capsid) of the NoV genome (*9*) and interrogation of the Norovirus Typing Tool database (www.rivm.nl/mpf/norovirus/typingtool). During November 2011–March 2012, the prevalence of sporadic NoV infections detected (in samples from newborns, infants, and children up to 5 years of age) by real-time reverse transcription PCR was 22.2% (121/545). A subset (≈50%) of the NoV-positive samples representative of the whole winter period was selected for sequence analysis, and 48 were successfully characterized in region A and region C. 

Among these 48 NoV strains, 20 (41.7%) were characterized as the variant GII.4 New Orleans 2009, a smaller number, 6 (12.5%), displayed a New Orleans 2009 polymerase (pol) but 2 distinct GII.4 capsid sequences, which were not typeable in the Norovirus Typing Tool database, and only 2 (4.2%) GII.4 strains of the variant Den Haag 2006b were detected. Moreover, 4 sporadic cases in November 2011 and January 2012 and a small outbreak in February 2012 were related to a GII.Pe_GII.4 recombinant strain. After the set of sequences of GII.4 variants from the Norovirus Typing Tool database was updated (access to the updated database: April 11, 2013), 5 (10.4%) GII.Pe_GII.4 recombinant strains were characterized as variant Sydney 2012.

From April through October 2012, a total of 56 (7.6%) NoV-positive samples were detected from 737 analyzed samples, of which 34 (60.7%) NoV-positive samples could be sequenced. Of these, 41.2% were characterized as GII.3 (mostly with a GII.Pb pol), 26.5% as GII.Pg_GII.1, and 17.6% as GII.4 variants. From spring to fall 2012, the variant New Orleans 2009 became the predominant GII.4 strain, and the variant Sydney 2012 strain apparently disappeared. 

During November–December 2012 and January 2013, ISGEV detected NoV infection in 90 (28.9%) of 311 children hospitalized for gastroenteritis. This finding is comparable to a prevalence of 25.2% in the same period (November–January) of the 2011–12 winter season. A representative subset of 45 samples was randomly selected for sequencing, and 26 (74.3%) of 35 fully typed strains were characterized as GII.4 Sydney 2012, which suggested that the new NoV variant had become the predominant strain in Italy. 

Our findings seem to mirror observations of a report from Denmark that documented the onset and circulation at low prevalence of the variant GII.4 Sydney 2012 at the beginning of 2012 with a marked increase in the prevalence only by the end of 2012 (*10*). Our surveillance detected the emergence of this variant in Italy at the end of 2011 and provided us with one of the earliest strains of the variant GII.4 Sydney 2012. This novel variant has a common ancestor with the NoV GII.4 variants Apeldoorn 2008 and New Orleans 2009 and has several amino acid changes on the main epitope in the capsid P2 domain (*10*). 

Sequence analysis of these early strains of the GII.4 variant Sydney 2012 could help clarify the mechanisms driving its global emergence and spread. Continued surveillance for NoV infections through ISGEV and additional data on clinical and epidemiologic features will enable further assessment of the public health implications of the new variant GII.4 Sydney 2012 in Italy.
